# Microcirculatory and Rheological Adaptive Mechanisms at High Altitude in European Lowlander Hikers and Nepalese Highlanders

**DOI:** 10.3390/jcm12082872

**Published:** 2023-04-14

**Authors:** Paolo Salvi, Andrea Grillo, Fausto Brunacci, Francesca Severi, Luca Montaguti, Sylvie Gautier, Lucia Salvi, Enzo Pretolani, Gianfranco Parati, Athanase Benetos

**Affiliations:** 1 Department of Cardiology, IRCCS, Istituto Auxologico Italiano, 20149 Milan, Italy; 2 Department of Medical, Surgical and Health Sciences, University of Trieste, 34100 Trieste, Italy; 3 Department of Emergency, Internal Medicine and Cardiology, Internal Medicine, ‘M. Bufalini’ Hospital, 47521 Cesena, Italy; 4 CHRU-Nancy, Pôle “Maladies du Vieillissement, Gérontologie et Soins Palliatifs”, Université de Lorraine, 54800 Nancy, Francea.benetos@chru-nancy.fr (A.B.); 5 Medicina II Cardiovascolare, AUSL-IRCCS di Reggio Emilia, 42122 Reggio Emilia, Italy; lsalvi.md@gmail.com; 6 Department of Medicine and Surgery, University of Milano-Bicocca, 20126 Milan, Italy; 7 DCAC u1116, INSERM, Université de Lorraine, 54000 Nancy, France

**Keywords:** acute mountain sickness, blood viscosity, cardiovascular risk, chronic mountain sickness, erythrocyte deformability, haemorheology, microcirculation, high altitude

## Abstract

Background: Physical activity at high-altitudes is increasingly widespread, both for tourist trekking and for the growing tendency to carry out sports and training activities at high-altitudes. Acute exposure to this hypobaric–hypoxic condition induces several complex adaptive mechanisms involving the cardiovascular, respiratory and endocrine systems. A lack of these adaptive mechanisms in microcirculation may cause the onset of symptoms of acute mountain sickness, a frequent disturbance after acute exposure at high altitudes. The aim of our study was to evaluate the microcirculatory adaptive mechanisms at different altitudes, from 1350 to 5050 m a.s.l., during a scientific expedition in the Himalayas. Methods: The main haematological parameters, blood viscosity and erythrocyte deformability were assessed at different altitudes on eight European lowlanders and on a group of eleven Nepalese highlanders. The microcirculation network was evaluated in vivo by conjunctival and periungual biomicroscopy. Results: Europeans showed a progressive and significant reduction of blood filterability and an increase of whole blood viscosity which correlate with the increase of altitude (*p* < 0.02). In the Nepalese highlanders, haemorheological changes were already present at their residence altitude, 3400 m a.s.l. (*p* < 0.001 vs. Europeans). With the increase in altitude, a massive interstitial oedema appeared in all participants, associated with erythrocyte aggregation phenomena and slowing of the flow rate in the microcirculation. Conclusions: High altitude causes important and significant microcirculatory adaptations. These changes in microcirculation induced by hypobaric–hypoxic conditions should be considered when planning training and physical activity at altitude.

## 1. Introduction

Physical activity at high altitudes is increasingly widespread, for tourist trekking and for professional reasons (tour operators, mountaineers, athletes), as well as for the increasingly frequent choice to carry out sports activities and training at high altitudes. Moreover, everyday cable cars, cable railways and chair lifts allow several thousands of subjects, including elderly individuals and subjects with known or subclinical cardiovascular disease, to easily access high-altitude locations. This implies that a significant number of individuals are exposed to the potentially adverse cardiovascular effects induced by acute exposure to acute hypobaric hypoxia at high altitude, involving cardiovascular, respiratory and endocrine systems. Multiple adjustments to hypoxia are commonly associated with histological and ultrastructural changes in the various tissues of the human body [1].

In acute exposure to high altitudes, the loss or failure to develop adaptive skills can cause several symptoms grouped under the name of acute mountain sickness (AMS): headache, anorexia, asthenia, nausea, vomiting, dizziness, insomnia and easy irritability [2]. In the context of high-altitude clinical disorders, severe and life-threatening clinical conditions can also occur, as high-altitude pulmonary oedema (HAPO) and high-altitude cerebral oedema (HACO). The incidence and the seriousness of the symptoms seem to depend on the speed of the ascent, the altitude reached and mainly on the individual predisposition [3,4].

There is much misunderstanding about the pathology of AMS. Many areas of high-altitude pathology remain controversial, and the needs and opportunities for further research in the field are great. Several studies have been performed to evaluate pulmonary, cardiac and vascular adaptations to altitude. Nevertheless, the adaptive mechanisms of the arteriolar-capillary system are not clearly understood yet.

An adequate blood supply in relation to the needs of the different tissues and organs is guaranteed by the activity of the small arteries and arterioles, which play a key role in maintaining microcirculatory homeostasis. In addition to the functional activity of the arterioles, the haemorhelogical properties of the blood, i.e., its viscosity, erythrocyte deformability and erythrocyte aggregability phenomena, must also be considered in a global evaluation of the microcirculation.

The aim of our research was to study the changes in the microcirculatory system induced by acute exposure to high altitude. A scientific expedition to the Himalayas was organised for this purpose, comparing the microcirculatory response to hypobaric hypoxia at high altitudes in a group of white European lowlanders and Nepalese highlanders.

## 2. Materials and Methods

### 2.1. Participants

The participants in this scientific expedition were recruited from a group of white European lowlanders, permanently living almost at sea level (<200 m a.s.l.), and a group of Rai Nepalese highlanders, permanently residing in the Khumbu valley between 3400 and 4930 m above sea level sea (a.s.l.) (mean ± SD: 4007 ± 583 m a.s.l.).

The presence of chronic disease was considered an exclusion criterion to be involved in the study. No chronic treatments were taken by the study participants, and no drugs were taken during the high-altitude ascension.

### 2.2. Protocol of the Study

After a 3-day stay in Kathmandu (Nepal, 1350 m a.s.l.), the European lowlanders flew to Lukla airport (2840 m a.s.l.), starting a trek to reach the village of Namche Bazaar (3400 m a.s.l.) the next day. Here, they stayed for 2 full days for acclimatisation. From Namche Bazaar, the two groups of lowlanders and highlanders walked together for 3 days finally reaching Lobuche (5050 m a.s.l.), at the “Pyramid International Laboratory” of the Italian National Research Council (CNR), on the southern slope of Everest.

Data were collected at Kathmandu and Namche Bazaar in hotel rooms and at Lobuche in the Pyramid International Laboratory. Barometric pressure and ambient temperature were recorded at the time of each study. Examinations were performed in the morning after at least 18 h of physical rest and after a stay of at least 1 h in a room with a constant temperature of 19 ± 1 °C.

**Figure 1:**
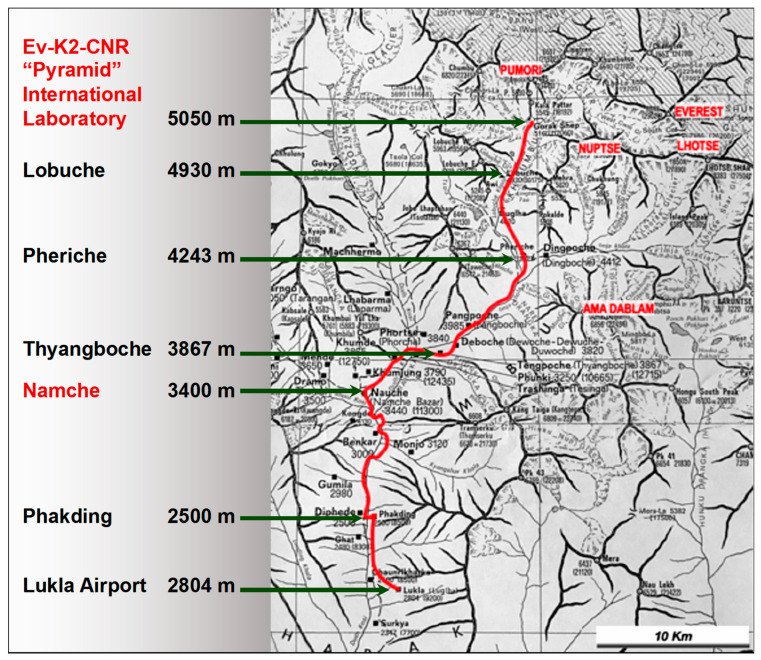
Trekking from Lukla airport (2804 m above sea level) to the international high-altitude research laboratory Ev-K2-CNR, “Pyramid” (5050 m a.s.l.). The tests were performed in Namche Bazar (3400 m a.s.l.) and at the “Pyramid” laboratory.

European lowlanders were studied at different altitudes (Figure 1):

- At 1350 m a.s.l., on the second day of permanence in Kathmandu;
- At 3400 m a.s.l., on the second day of permanence in Namche Bazar;
- At 5050 m. a.s.l., on the second day of permanence in Lobuche;
- And the 8th day of stay in this high-altitude laboratory.

Nepalese highlanders were studied on the same day as Europeans at 3400 m a.s.l. (Namche Bazar) and at 5050 m a.s.l. (Lobuche).

### 2.3. Complete Blood Count

Complete blood count was performed with the Coulter counter (Coulter Microdiff 18, Beckman Instruments Inc., Miami, FL, USA), assessing white blood cell (WBC) count and their distribution, red blood cell (RBC) count, haemoglobin (Hb), haematocrit (Hct), mean corpuscular volume (MCV), mean corpuscular haemoglobin concentration (MCHC), red cells distribution wave (RDW), platelet (PLT) count and plateletcrit (Pct). Blood was drawn in the morning, after a 10 min resting period and in a seated position. Sampling was done within 60 s after the application of tourniquet, using sterile procedures, 21 G needles and vacuum tubes containing EDTA-2K^+^.

### 2.4. Blood Viscosity

Whole blood viscosity was measured at native Hct and at different shear rates (450, 225, 90, 45 and 22 s^−1^), using a DV-II+ Cone/Plate Viscometer Brookfield (Brookfield Engineering Laboratories, Inc., Stoughton, MA, USA), according to the guidelines for haemorheological laboratory techniques [5], with Haake D8 Thermostat (Haake, Karlsruhe, Germany), in order to maintain the sample at a stable temperature of 37 °C throughout the test. Blood viscosity values at 37 °C are expressed in milliPascal × seconds (mPa∙s) [5].

Plasma viscosity was measured by the Viscometer Brookfield at 225 s^−1^, 37 °C, on plasma obtained by centrifuging blood at 2000× *g* for 15 min.

### 2.5. Blood Filterability

Erythrocyte deformability was assessed by the whole blood filterability method developed by Reid and Dormandy [6]. This filtration test measures the time required by 1 mL of blood to pass through membranes crossed by micropores of 5 µm in diameter and 12.5 µm in length (Nucleopore Co., Pleasanton, CA, USA) under a negative pressure of 20 cm H_2_O. Filtration time was measured in triplicate, and the mean value was recorded. Data were expressed as volume of red blood cells (VRBC) filtered per minute, corrected by the haematocrit, as follows:

VRBC = 60/filtration time (s) × Hct.

### 2.6. In Vivo Microcirculatory Study

Periungual capillaroscopy and conjunctival biomicroscopy were performed by means of an SV8 Zeiss video-stereomicroscopy (Carl Zeiss, Oberkochen, Germany) with cold light source Schott KL 1500 (Schott Glaswerke, Wiesbaden, Germany) and Panasonic S-VHS recording equipment (Panasonic Corporation, Osaka, Japan). The analysis of the flows and the morphological characteristics of the microcirculatory network was performed blindly by two different operators, not participating in the scientific expedition.

Periungual capillaroscopy evaluated the periungual capillaries at the 2nd, 3rd, 4th and 5th fingers in both hands. The following parameters were mainly considered:

- Morphology of the capillary loops: normally the capillary loops have a “hairpin” appearance, the afferent branch being thinner than the efferent branch, the latter being slightly sinuous. The presence of capillaries characterised by repeated curves and twists (kinking) of ectasias and abnormal dilations is reported.
- Presence of pericapillary oedema: normally the capillary loops are well defined, and the blood flow is evident and easy to analyse. In the presence of pericapillary oedema, the definition of the capillary loops is reduced. A soft-focus effect (“flou effect”) is therefore observed on biomicroscopy, in which the contours of the capillary loops are less defined. This soft-focus effect can also be heavy, and in this case the loops appear poorly defined. Severe degrees of oedema do not allow visualisation of the capillary loops.
- Blood flow assessment: normally on biomicroscopy, the flow appears continuous, with regular and periodic variations in the flow velocity depending on the vasomotor activity, in particular by the slow wave of vasomotion [7]. Functional alterations of flow can be documented by the presence of interruption of the flow, sometimes followed by the inversion of the flow. In the presence of erythrocyte aggregates, the flow is discontinuous, with the progression of red blood cells in packets.

Conjunctival biomicroscopy evaluated the microcirculatory network in the bulbar conjunctiva. In each study participant, three areas in the observation field of the bulbar conjunctiva were selected, in order to control the same areas at different altitudes. The presence of irregularities in the calibre of the microvessels, hyperaemia or rarefaction of the microcirculatory network and alterations in the blood flow are reported.

### 2.7. Oxygen Saturation

Arterial oxygen saturation (SaO_2_) was measured by means of 7845 Pulse Oximeter Kontron (Kontron GmbH, Ismaning, Germany) with finger clip sensor.

### 2.8. Haemodynamic Parameters

Heart rate and blood pressure were measured using a validated and calibrated sphygmomanometer. Three measurements were recorded, and the average of the second and third was retained.

### 2.9. Statistical Analysis

Results are expressed as median and interquartile range. Normal distribution of variables was assessed by Shapiro–Wilk test. Differences between two groups (Europeans and Nepalese) for all variables were evaluated with Student’s t-test for unpaired data or with independent samples Mann–Whitney U test for variables not normally distributed. Levene’s test was used to assess equality of variances. Statistical analysis of parameter’s alterations with altitude was calculated using Friedman’s test and the subsequent two-tailed Wilcoxon test for non-parametric paired data. Statistical analysis was performed by using the Statistical Package for the Social Sciences (SPSS for Windows, Release 20.0; SPSS, Chicago, IL, USA). A *p* value less than 0.05 was considered as significant.

## 3. Results

Eight European participants in the scientific expedition (six men and two women) and 11 male Nepalese porters agreed to participate in the study. Their main anthropometric characteristics are shown in Table 1

Oxygen saturation significantly decrease at 3400 (*p* < 0.02) and 5050 m a.s.l. both in European lowlanders (Friedman *p* value < 0.001) and Nepalese highlanders (*p* < 0.001). At 5050 m a.s.l., SaO_2_ was significantly higher in highlanders than in lowlanders (*p* < 0.05).

Haematological and haemorheological changes with altitude in European lowlanders and Nepalese highlanders are shown in Table 2. After a first significant reduction at medium altitude (*p* < 0.05 at 3400 m a.s.l.), RBC and PLT significantly increased above 5000 m in lowlanders, with an even more significant increase after an eight-day stay (*p* < 0.05).

The Hct values also decreased at 3400 m a.s.l. and then increase at high altitude, with a significant increase after 8 days of stay (*p* < 0.05) at 5050 m a.s.l. At 3400 m a.s.l., highlanders had significantly higher RBC, HB and Hct values than European lowlanders. At the highest altitude, these parameters did not significantly change in highlanders, while they increased in lowlanders.

As a result, at 5050 m a.s.l. there were no longer any significant differences between these ethnic groups. Highlanders had significantly higher WBC and PLT values than lowlanders at both 3400 m a.s.l. that at 5050 m a.s.l. Hb tends to increase after the arrival at 5050 m., with a further increase after a stay at this altitude (*p* < 0.05). RDW tends to increase steadily with altitude, reaching significance levels at 5050 m a.s.l. (*p* < 0.05). MCV does not present any change.

**Figure 2:**
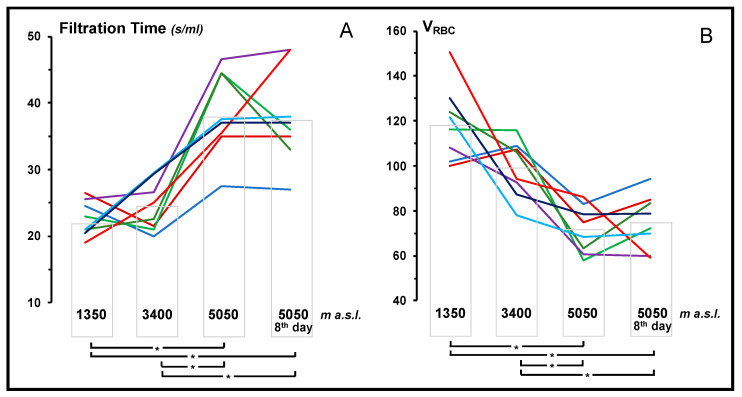
Blood Filterability in European lowlanders at different altitude. A: Filtration time required by 1 mL of whole blood to pass through 5 µm pore-membranes under a negative pressure of 20 cm H_2_O. B: volume of red blood cells filtered per minute, corrected by the haematocrit (VRBC = 60/filtration time (s) × Hct). a.s.l. = above sea level. * *p* < 0.001.

In European lowlanders, a progressive reduction in blood filterability significantly correlated (*p* < 0.001) with the increase in altitude was observed (Figure 2). Low values of blood filterability were also observed in the Nepalese porters already at 3400 m. (significantly lower than Europeans at the same altitude, *p* < 0.05). In highlanders, blood filterability did not increase between 3400 and 5050 m a.s.l., so Europeans and highlanders showed no difference at this latter altitude.

No change in plasma viscosity was observed at high-altitude in lowlanders, while the viscosity values of whole blood, after a significant transient reduction at 3400 m a.s.l. (*p* < 0.05), increased significantly to 5050 m, with a further increase after a stay at this altitude (a highly significant trend for all share rates). A significant increase in whole blood viscosity between 3400 m and 5050 m a.s.l. was also observed in highlanders (*p* < 0.05).

The Nepalese porters had a significant increase in the whole blood viscosity values from 3400 m to 5050 m a.s.l. (*p* < 0.05), at low and high share rates. At both these altitudes, blood viscosity was consistently higher in highlanders than in Europeans (*p* < 0.05), especially at high share rates.

Heart rate, systolic and diastolic blood pressures did not show significant changes with altitude in both lowlanders and highlanders (Table 3).

Appearance of pericapillary oedema at high altitude was a phenomenon recorded in all participants at this study, both lowlanders and highlanders (Table 4). Due to the massive oedema, the analysis of the capillary loops was not possible in a lowlander and a highlander at 3400 m a.s.l. and in two other lowlanders at 5050 m a.s.l.

Dilated capillary loops were observed in 80% of highlanders at 3400 m a.s.l. and no significant morphological variations of the periungual microcirculation were observed upon reaching 5050 m a.s.l. in this ethnic group. A slow and intermittent flow was observed in all lowlanders and in 50% of highlanders at 5050 m a.s.l.

Improved perfusion and generalised dilation of the microcirculation were observed on conjunctival biomicroscopy at 5050 m a.s.l. in European lowlanders and in almost all highlanders (Table 5). Two highlanders showed reduced arteriolar calibre and slight reduced perfusion.

## 4. Discussion

To our knowledge, this is the first study aimed at evaluating changes in all major microcirculatory and haemorheological parameters related to altitude variation in both the lowlander and highlander populations. This study highlighted evident changes induced by acute exposure to hypoxic–hypobaric high altitudes: increase in whole blood viscosity, reduction of red blood cell deformability, appearance of erythrocyte aggregation phenomena, slowing of the flow rate in the microcirculation and constant appearance of massive interstitial oedema, in addition to the already well-known increase in haemoglobin and haematocrit related to staying at high altitude. 

### 4.1. Haemorheological Changes with Altitude

The haemorheological characteristics depend on the properties of the blood components and their interaction. Moreover, blood, being a non-Newtonian fluid, has an inconstant viscosity depending on its speed inside the different vessels [8]. This depends on the shear rate, a gradient of velocity which exists among the several ideal flux floors, running one next to the other creating a tangential stress (shear stress). The absolute viscosity of a fluid depends on the shear stress/shear rate ratio. Shear rate is maximum at the capillary level, where viscosity seems to depend mainly on the red blood cells deformability. Low shear rate values are present in the veins where red blood cells aggregation is predominant. Medium shear rate values are present in the arteries.

In our study, a significant increase in whole blood viscosity, especially at high and medium shear rates, was observed in European lowlanders at very high altitude compared to baseline values recorded at 1300 m a.s.l. A significant and constant reduction in blood filterability with increasing altitude has also been documented in this European population.

At 3400 m a.s.l., Nepalese highlanders had increased basal blood viscosity and reduced whole blood filterability compared to Europeans both at the same altitude and at 1300 m a.s.l. At very high altitudes (5050 m a.s.l.), no significant worsening of these parameters was observed in highlanders, and there was no statistically significant difference between the two ethnicities. Therefore, it must be deduced that in Europeans the alterations that have been observed occur in a very acute way with very little possibility of compensation. On the other hand, since these alterations were constantly present in the Nepalese people, they are likely associated with compensatory phenomena and are thus better tolerated.

The main factors affecting blood viscosity are (i) plasma viscosity which mainly depends on fibrinogen and other proteins with high molecular weight, (ii) white blood cells count and deformability, (iii) Hct, (iv) red blood tendency to aggregate and (v) red blood cells deformability. The latter factor represents the ability of erythrocytes to change shape in response to external stresses.

Whole blood filterability (expressed as VRBC), assessed by the Reid and Dormandy’s filtration test, can be considered an indirect method to estimate erythrocyte deformability. Actually, other factors can affect VRBC, such as plasma viscosity, Hct, erythrocyte aggregability, WBC count and deformability. However, considering that the plasma viscosity does not change with altitude and that VRBC is a parameter already adjusted for Hct, the reduction of this parameter may be reasonably referred to a reduction in erythrocyte deformability.

This property allows individual red cells, whose mean resting diameter is 7–8 µ, to traverse nutritive capillaries with diameter between 3 and 5 µ [9]. Red blood cells deformability depends on several mechanisms, most of all biochemical, at the cytoplasm and cell-wall levels [10]. Four main factors affect erythrocyte deformability: (i) the red blood cells morphology, (ii) the surface/volume ratio, (iii) the wall flexibility and (iv) the internal viscosity. These connected and interdependent factors are influenced by several situations and by high-altitude hypobaric hypoxia. Haemorheological changes due to acidosis and ischemia are already well documented in the literature [11,12,13,14].

Our results agree with the studies by Mao et al. [15] performed in a climate-controlled normobaric hypoxic chamber, at O_2_ concentrations corresponding to an altitude of 2733 m a.s.l. This research group demonstrated a reduction in the volume and deformability of human erythrocytes induced by hypoxic exercise. Conversely, conflicting results come from studies performed on laboratory animals [16,17]. Effects of living and training at different altitudes on red blood cell deformability and erythrocyte aggregation were studied by Bor-Kucukatay et al. in Sprague Dawley rats [16]. The deformability of red blood cells in rats living and training at altitude was higher than that of rats living and training close to sea level and living at altitude and training close to sea level. In the authors’ opinion, this would be an adaptive mechanism to contribute blood flow in response to hypoxia at low shear stresses.

At 3400 m a.s.l., a transient decrease in blood viscosity was observed in lowlanders. This event may be partly due to the reduction of Hct and red blood cells, probably secondary to a phenomenon of fluid retention and a redistribution of intra- and extravascular fluid. These phenomena constitute a first stage of the physiological adaptations of altitude. An increase in Hct and in the number of red blood cells after staying at 5050 m a.s.l. was expected.

The results of our study agree with the findings of previous studies. A significant change in blood viscosity was also shown by Ernst et al. in lowlanders after acute exposure at moderately high altitude (2700 m a.s.l.) [18]. Lower values of whole blood viscosity were observed in lowlander populations compared to highlanders, permanently living at 3800 and 5100 m a.s.l. [19]. The results of the UBC-Nepal Expedition showed different blood viscosity behaviour at high altitudes in lowlanders and highlanders [20]. While the lowlanders showed a significant increase in whole blood viscosity with increasing altitude (from 1300 to 5050 m a.s.l.), in contrast, a group of Sherpa highlanders showed no change in blood viscosity at high altitudes. In this study, blood viscosity was measured only at high shear rate (225 s^−1^) [20].

### 4.2. Microcircolatory Changes with Altitude

The in vivo analysis of the periungual microcirculation showed the constant appearance of interstitial oedema even at modest altitudes (3400 m a.s.l.) both in lowlanders and in highlanders. The oedema then became particularly pronounced at very high altitude. A slowdown of the capillary flow was shown at high altitudes, in some cases characterised by the appearance of frequent flow stops (intermittent flow). On the other hand, a vasomotion increase was recently described in the lowlanders at high altitudes and could be explained as a compensation attempt in order to permit an adequate tissue perfusion [7]. This vasomotion increase was probably due to an increased adrenergic activity, as attested by high norepinephrine levels [7]. Vasomotion activation phenomenon in the native people was similar to those registered in the Europeans at the same altitude; therefore, it may be inferred that the modifications which were observed in the Europeans are chronically present in the natives. On the other hand, all the modifications which were observed on the first day after the arrival at 5050 m a.s.l., were more marked after eight days [7].

Similar results were achieved during the Xtreme Everest 2 expedition using combined laser Doppler fluximetry and white light spectroscopy in 77 lowlanders and 56 Nepalese highlanders [21,22]. When exposed to hypobaric hypoxia at Everest base camp (5300 m a.s.l.), highlanders demonstrated superior preservation of their peripheral microcirculatory perfusion with sustained microvascular reactivity with enhanced vasomotion when compared with lowlanders [21]. More in-depth analyses of laser-Doppler signals [22] performed on a subset of participants suggested that the increased variability in the microcirculatory blood flow signal seen in Nepalese highlanders was indicative of a beneficially enhanced microcirculatory adaptive capacity through more effective autoregulation within the microvascular network. The results of this scientific expedition suggest that peripheral tissues play an important physiological role in cardiovascular adaptation to hypoxia and that this role is better developed in native highlanders than in lowlanders.

European lowlanders had a regular calibre of capillary loops. The periungual microcirculation in the highlanders was instead characterised by the constant presence of dilated capillary loops in both the evaluated altitude. This phenotypic aspect could be considered as a chronic adaptation to a hypoxic–hypobaric condition. As confirmation of this adaptive process, at the level of the bulbar conjunctiva the microcirculation of the Nepalese highlanders was characterised by the presence of dilated capillaries, repeated curves and twists and by dilatation of the arteriolar network. Aspects of improvement in microcirculatory perfusion and the presence of dilated arterioles are evident in all lowlanders at high altitudes, as a likely sign of acute adaptation to hypoxia.

Changes in haemodynamic parameters do not appear to be relevant for the interpretation of the haematological and rheological results, since no significant changes were recorded in heart rate and blood pressure in both lowlanders and highlanders.

The in vivo study of the bulbar conjunctiva documented improvement in perfusion and generalised dilatation of the microcirculation at 5050 m a.s.l. both in the lowlanders and in the highlanders. Our data agree with the results of other studies investigating the changes induced by high altitude on the sublingual microcirculatory network [23,24,25,26,27]. Hilty et al. [23] during the 2013 Swiss High Altitude Medical Research Expedition to Mount Himlung Himal, Nepal (7126 m), showed a significant increase in total vessel density after a total of 3 weeks of altitude exposure. Data were collected at 6022 m a.s.l. in 36 healthy volunteers and at 7042 m a.s.l. in 10 summiteers. In this study, the recruitment of total sublingual capillary density associated with high altitude occurred despite decreased cardiac output, unchanged blood pressure and systemic vascular hindrance, thus appearing as an intrinsic compensatory mechanism of the microcirculation to increase oxygen extraction capacity [23]. Previously, two high-altitude studies by Martin et al. [25,26], evaluating the changes of the sublingual microcirculation with altitude, were unable to detect an increase in capillary density. A first study was performed during a mountaineering expedition to Cho Oyu (8201 m) in the Himalayas [25]. Data were collected on healthy volunteers at 4900 m (n: 12), 5600 m (n: 9) and 6400 m a.s.l. (n: 4). This study showed a significant reduction in microcirculatory flow index at high altitude (4900 m a.s.l.) when compared with baseline in small- to medium-sized (<50 µm) blood vessels, with a further reduction in microcirculatory flow index within these microvessels at extreme altitudes (6400 m a.s.l.). A second study was performed during an expedition to Mount Everest (8849 m) [26]. Data were collected at 3500 m (n: 24), 5300 m (n: 24), 6400 m (n: 14) and 7950 m a.s.l. (n: 4). As in the previous expedition of this research group, the microcirculatory flow index was significantly reduced at high altitudes in vessels < 50 µm. This study also showed no correlation between microcirculatory flow index and vessel density at any altitude. In fact, at 5300 m a.s.l. the total vessel density significantly increased, particularly in medium-size microvessels (25–50 µm) [26]. The ascent to high altitudes would therefore lead to a reduction of the sublingual microcirculatory flow and to an increase in the density of the capillaries. Although the examinations at extreme altitudes were performed in uncontrolled conditions, in low temperatures and variable weather conditions, a strength of these high-altitude scientific expeditions was the possibility to evaluate the sublingual microcirculation at these exceptional altitudes. The Xtreme Everest 2 research expedition [27] was conducted on healthy volunteers ascending to Everest base camp (5300 m a.s.l.): 63 Nepalese highlanders and 68 European lowlander volunteers. This study demonstrated differences between highlander and lowlander sublingual microcirculatory responses to hypobaric–hypoxic conditions at high altitude. Microcirculatory blood flow and capillary density did not differ between cohorts at baseline. At high altitude, lowlanders experienced decreased blood flow but increased capillary density, whereas Nepalese highlanders showed a higher sublingual microcirculatory blood flow and greater capillary density than lowlanders, supporting the notion that the peripheral microcirculation plays a key role in the process of long-term adaptation to hypoxia [27].

West proposed that the pathogenetic mechanism of high-altitude pulmonary oedema is stress failure of pulmonary capillaries [28]. This results in a high permeability form of oedema with the escape of high molecular weight proteins and blood cells into the alveolar spaces. In addition, the basement membrane of the endothelial layer is frequently exposed, and this highly reactive surface attracts and activates platelets and neutrophils. The results are the development of small thrombi which are frequently seen in the pulmonary oedema. Hypoxic pulmonary vasoconstriction raises the pressure in some capillaries because the constriction is uneven. Moreover, hypobaric hypoxia brings about muscularisation of the most peripheral portions of the pulmonary arterial tree and involves the smallest pulmonary arteries, the pulmonary arterioles and the precapillary vessels [29]. This peripheral muscularisation becomes associated with a raised pulmonary arterial pressure that is moderate in infants and children [30,31] and slight in adults [32]. It is slowly reversible on descent to sea level.

There is far more to the pathology of this disease than the collection of oedematous fluid in the lung parenchyma. There is a strong thrombotic element, so multiple pulmonary thromboses and areas of red infarction occur [33]. The same thrombotic tendency is seen in the cerebral circulation, so the oedema and petechial haemorrhages in the brain are frequently associated with thrombosis in the cerebral venous sinuses. A combination of altered activity of blood platelets, blood coagulation factors and a breakdown of fibrinolytic system is believed to be responsible for intravascular sludging of erythrocytes and for the formation of fibrin thrombi not only in the cerebral and pulmonary circulation but also in the glomerular capillaries of the kidneys [34].

### 4.3. Limitations of the Study

The main limitation of our study was the relatively small number of enrolled participants. However, the limit conditions in which the researchers worked must be considered. Materials were carried on back by Nepalese porters and yaks (therefore the weight was limited to small amounts); the energy supply was scarce. In Namche, the electric power was available only for few hours a day, and sometimes it was unsteady.

Regarding the in vitro estimation of erythrocyte deformability, actually the red cell deformability should be more correctly evaluated by means of proper filterability methods, with washing and resuspension of the standard haematocrit erythrocytes, but the conditions in which the test were carried out must be firstly taken into consideration. Furthermore, we believe that other methods which use centrifuge, red cell washing and resuspension cause such and so many traumas to the erythrocyte that the test data may not mirror the real deforming ability of the erythrocyte, especially when modifications linked to hypoxia are studied.

It should bet be also pointed out that some instruments are not suitable to work at high altitudes, not only for calibration problems (for example, no instruments capable of evaluating reliably the O_2_ and CO_2_ transcutaneous pressure above 3500 m a.s.l. were available) but also because within some instruments air bearings may prevent their usage at high altitudes with low atmospheric pressure. For this reason, the choice of devices was often forced by the critical conditions of the study.

## 5. Conclusions

These data demonstrate how altitude determines important and significant microcirculatory modification, with haematological and haemorheological alterations. At high altitudes, we verified an increase of blood viscosity, associated with a red blood cell deformability reduction, an increased intravascular red blood cell aggregability and an increase of the haematocrit. We also observed signs of an altered capillary permeability. Nepalese highlanders present permanent and chronic haemorheological and haematological alterations, while in the Europeans these are acute and tend to worsen when staying at high altitudes.

These altitude-induced changes in microcirculation should be considered when planning training and physical activity at altitude. Further studies are needed to verify the etiopathogenetic role of microcirculatory alterations in the pathophysiology of altitude sickness. In particular, the haemorheological and microcirculatory alterations could explain some minor symptoms of acute mountain sickness and could play an important role among the causes of the anatomopathological alterations in course of high-altitude pulmonary and cerebral oedema.

## Figures and Tables

**Table 1:** Anthropometric characteristics of participants.

Parameter	Lowlanders	Highlanders	*p*-Value
Sex, m/f	6/2	11/0	
Age, years	34.6 ± 3.5	27.8 ± 6.6	0.017
Height, cm	173.4 ± 6.3	160.8 ± 7.7	0.002
Weight, Kg	76.8 ± 7.6	52.7 ± 5.6	<0.001
BMI, Kg/m^2^	25.5 ± 1.8	20.4 ± 1.8	<0.001
BSA, m^2^	1.91 ± 0.12	1.54 ± 0.11	<0.001

Data are shown as mean ± standard deviation. Significance is expressed by *p*-value. BMI = body mass index; BSA = body surface area, estimated by the Du Bois formula; f = females; m = males.

**Table 2:** Haematological and haemorheological changes with altitude in European lowlanders and Nepalese highlanders.

Parameters	Ethnicity	Altitude, m a.s.l.				Trend
		1350	3400	5050	5050 (8th Day)	*p*-Value
SaO_2_, %	Lowlanders	95.9 (95.4–97.0)	92.6 (89.0–94.5) *	80.5 (77.0–84.0) *	83.5 (77.8–89.5) *	<0.001
	Highlanders		94.0 (92.4–95.5)	85.0 (83.0–91.0) *^†^		
White Blood Cells,	Lowlanders	6.77 (6.29–7.11)	6.15 (5.77–6.39) *	6.32 (5.97–6.89)	7.30 (6.60–7.52)	0.391
×10^3^/μL	Highlanders		10.22 (8.66–11.64) ^†^	9.40 (7.11–10.00) ^†^		
Platelets, ×10^3^/μL	Lowlanders	258 (236–289)	239 (217–255) *	318 (290–358) *	327 (297–386) *	<0.001
	Highlanders		345 (283–365) ^†^	382 (338–451) ^†^		
Plateletcrit, %	Lowlanders	0.21 (0.18–0.24)	0.20 (0.18–0.22)	0.25 (0.25–0.27) *	0.26 (0.25–0.29) *	<0.001
	Highlanders		0.27 (0.21–0.29) ^†^	0.28 (0.27–0.31)		
Red Blood Cells, ×10^6^/μL	Lowlanders	4.82 (4.65–5.12)	4.48 (4.32–4.56) *	5.11 (4.71–5.27)	5.19 (4.90–5.38) *	0.040
	Highlanders		5.36 (5.01–5.55) ^†^	5.15 (4.89–5.50)		
Haemoglobin, g/dL	Lowlanders	14.6 (13.8–15.0)	14.1 (13.6–14.4)	15.5 (14.6–16.6) *	16.3 (15.3–16.9) *	0.002
	Highlanders		17.2 (16.2–17.6) ^†^	16.3 (15.8–16.8)		
Haematocrit, %	Lowlanders	43.7 (41.9–44.4)	39.5 (38.4–40.9) *	45.3 (42.8–48.0)	46.6 (43.4–48.5) *	0.055
	Highlanders		48.0 (46.0–50.1) ^†^	47.8 (45.0–48.7)		
MCV, fl	Lowlanders	90.1 (86.8–92.3)	89.2 (85.6–91.6)	89.5 (87.2–91.6)	88.8 (87.4–91.3)	0.650
	Highlanders		89.9 (88.5–93.6)	89.0 (88.4–94.2)		
MCH, pg	Lowlanders	30.0 (28.4–31.1)	31.6 (30.6–32.4) *	30.6 (30.4–31.5)	31.2 (30.2–32.2) *	0.139
	Highlanders		32.0 (31.2–33.3)	30.6 (30.1–32.7) *		
MCHC, g/dL	Lowlanders	32.9 (32.5–33.0)	35.3 (35.1–36.3) *	34.4 (33.9–34.8) *	34.9 (34.6–35.3) *	0.034
	Highlanders		35.4 (34.9–35.8)	34.4 (33.9–34.8) *		
RDW, %	Lowlanders	13.0 (12.8–13.6)	13.0 (12.6–13.8)	13.6 (13.3–14.3) *	14.2 (13.6–14.7) *	0.002
	Highlanders		13.4 (12.8–13.6)	13.5 (13.0–13.6)		
Blood Filtration Time,	Lowlanders	22.0 (20.6–25.2)	23.7 (21.1–28.6)	37.2 (35.1–44.5) *	36.5 (33.5–45.5) *	<0.001
s/mL	Highlanders		39.0 (37.9–62.9) ^†^	41.0 (34.0–56.7)		
Blood Filterability, V_RBC_	Lowlanders	113.7 (102.5–128.3)	100.0 (88.4–108.5)	71.5 (61.2–81.8) *	75.5 (62.2–84.6) *	<0.001
	Highlanders		72.8 (43.9–77.2) ^†^	67.7 (41.2–87.3)		
Blood Viscosity SR 450,	Lowlanders	3.87 (3.59–4.07)	3.24 (3.15–3.42) *	3.70 (3.64–4.03)	4.33 (4.08–4.67) *	0.008
mPa·s	Highlanders		4.08 (3.74–4.45) ^†^	4.29 (3.94–4.81) *^†^		
Blood Viscosity SR 225,	Lowlanders	4.50 (3.96–4.60)	4.12 (3.72–4.35)	4.74 (4.54–4.96) *	5.22 (4.93–5.45) *	<0.001
mPa·s	Highlanders		4.46 (4.29–4.60) ^†^	5.13 (4.70–5.32) *^†^		
Blood Viscosity SR 90,	Lowlanders	4.56 (4.47–4.76)	3.68 (3.43–3.95) *	4.72 (4.42–5.02)	5.28 (5.01–5.74) *	0.003
mPa∙s	Highlanders		4.83 (4.37–5.18) ^†^	5.02 (4.87–5.68) *^†^		
Blood Viscosity SR 45,	Lowlanders	4.19 (3.64–4.34)	3.18 (2.24–4.39)	7.92 (7.39–8.72) *	7.81 (5.35–5.96) *	<0.001
mPa∙s	Highlanders		5.42 (5.22–6.49) ^†^	7.43 (6.97–8.15) *		
Blood Viscosity SR 22,	Lowlanders	5.69 (5.36–5.96)	4.32 (3.03–4.46) *	6.12 (5.51–6.19)	6.49 (6.30–6.75) *	0.001
mPa∙s	Highlanders		4.53 (4.03–4.76)	6.14 (5.66–6.39) *		
Plasma Viscosity, mPa∙s	Lowlanders	1.49 (1.32–1.60)		1.50 (1.34–1.62)		

Data are shown as median (interquartile range). a.s.l. = above sea level. * *p* < 0.05, lowlanders at high-altitude versus 1350 m a.s.l., and highlanders at 5050 m versus 3400 m a.s.l.; ^†^ *p* < 0.05, highlanders versus lowlanders. MCH = mean corpuscular haemoglobin; MCHC = mean corpuscular haemoglobin concentration; MCV = mean corpuscular volume; RDW = red blood cells distribution width; SaO_2_ = arterial oxygen saturation; SR = shear rate. Whole blood Filterability is an index of erythrocyte deformability and is expressed as volume of red blood cells (VRBC) filtered per minute [VRBC = 60/filtration time × Hct].

**Table 3:** Heart rate and blood pressure changes with altitude in European lowlanders and Nepalese highlanders.

Parameters	Ethnicity	Altitude, m a.s.l.			
		1350	3400	5050	5050 (8th Day)
Heart Rate, b.p.m.	Lowlanders	67.2 (59.8–74.9)	71.2 (54.8–86.7)	78.3 (74.6–82.4)	76.5 (63.8–83.7)
	Highlanders		62.7 (57.0–69.7)	64.3 (60.7–79.0)	
Systolic Blood Pressure,	Lowlanders	112.7 (109.1–116.4)	118.5 (112.2–120.9)	119.2 (114.5–122.2)	121.0 (111.5–133.0)
mmHg mmHg *×* 10^3^/μL	Highlanders		115.0 (109.7–118.7)	118.3 (102.7–121.3)	
Diastolic Blood Pressure, mmHg	Lowlanders	66.7 (62.6–71.3)	68.0 (64.7–77.1)	70.5 (66.2–75.6)	70.5 (63.1–80.2)
	Highlanders		70.0 (66.7–76.0)	72.3 (70.7–78.7)	

Data are shown as median (interquartile range). a.s.l. = above sea level.

**Table 4:** Microvascular change with altitude. Periungual capillaroscopy.

**European Lowlanders**								
**N.**	Oedema *				**Flow and Morphology**			
	**1350 m**	**3400 m**		**5050 m**	**1350 m a.s.l.**	**3400 m a.s.l.**		**5050 m a.s.l.**
01	0	++		++	continuous flow	continuous flow		tortuous loops
02	0	+		+	continuous flow	very slow flow		very slow flow, with stops
03	0/+	+		++	slightly reduced flow	continuous flow		tortuous loops
04	0	+++		+++	continuous flow	not evaluable (oedema)		not evaluable (oedema)
05	0/+	+/++		+/++	slightly reduced flow	continuous flow		slow intermittent flow
06	0	0/+		+	continuous flow	slow flow		slow intermittent flow
07	0	+		+++	continuous flow	continuous flow		not evaluable (oedema)
08	0/+	+/++		++/+++	continuous flow	slow flow		not evaluable (oedema)
**Nepalese Highlanders**								
**N.**	Oedema *				**Flow and Morphology**			
	**3400 m**		**5050 m**		**3400 m a.s.l.**		**5050 m a.s.l.**	
01	0/+		+		dilated loops		low flow	
02	0		+		slow flow, dilated loops		slow flow, dilated loops	
03	+		++		continuous flow		continuous flow	
04	+++		+++		not evaluable (oedema)		not evaluable (oedema)	
05	0		++		very slow flow, dilated loops		very slow flow	
06	++		+		continuous flow, dilated loops		continuous flow, dilated loops	
07	+		+/++		continuous flow, dilated loops		continuous flow, dilated loops	
08	++		++		continuous flow, dilated loops		continuous flow, dilated loops	
09	+		++		continuous flow, dilated loops		continuous flow, dilated loops	
10	+		++		continuous flow, dilated loops		slow flow, dilated loops	
11	0		0/+		slow flow		slow flow	

* 0, no oedema; +, slight soft-focus effect; ++, heavy soft-focus effect; +++, severe oedema (the capillary loops are not clearly evident). a.s.l. = above sea level.

**Table 5:** Microvascular change with altitude. Conjunctival biomicroscopy.

**European Lowlanders**				
**N.**	**Flow and Morphology**			
	**1350 m a.s.l.**	**3400 m a.s.l.**		**5050 m a.s.l.**
01	regular morphology and flow	dilation and improved perfusion		dilation and improved perfusion
02	irregular calibre of venules	arteriolar constriction		dilation and improved perfusion
03	regular morphology and flow	slight reduction of the overall flow		dilation and improved perfusion
04	n.d.	n.d.		n.d.
05	regular morphology and flow	dilation and improved perfusion		dilation and improved perfusion
06	arteriolar and capillary rarefaction	slight reduction of the overall flow		dilation and improved perfusion
07	regular morphology and flow	regular morphology and flow		dilation and improved perfusion
08	slightly granular flow	slight reduction of the overall flow		dilation and improved perfusion
**Nepalese Highlanders**				
**N.**	**Flow and Morphology**			
	**3400 m a.s.l.**		**5050 m a.s.l.**	
01	n.d.		n.d.	
02	granular flow, arteriolar rarefaction, kinking *		slight dilation in the arteriolar network	
03	regular flow and arteriolar morphology, kinking *		slight dilation in the arteriolar network	
04	regular flow and arteriolar morphology, kinking *		reduced arteriolar calibre	
05	regular flow and arteriolar morphology, kinking *		n.d.	
06	regular flow, slight arteriolar reduction, kinking *		slight reduction of the overall flow	
07	regular flow and arteriolar morphology, kinking *		slight dilation and improved perfusion	
08	regular flow and arteriolar morphology, kinking *		arteriolar dilation and improved perfusion	
09	regular flow and arteriolar morphology, kinking *		slight dilation and improved perfusion	
10	regular flow and arteriolar morphology, kinking *		arteriolar dilation and improved perfusion	
11	regular flow, transient arteriolar spasms		arteriolar dilation and improved perfusion	

* Kinking: capillaries characterised by repeated curves and twists. a.s.l. = above sea level.

## Data Availability

The data presented in this study are available upon reasonable request from the corresponding author. The data are not publicly available due to privacy concerns.
